# Isolation of SARS-CoV-2 strains carrying a nucleotide mutation, leading to a stop codon in the ORF 6 protein

**DOI:** 10.1080/22221751.2021.1884003

**Published:** 2021-02-18

**Authors:** Serena Delbue, Sarah D’Alessandro, Lucia Signorini, Maria Dolci, Elena Pariani, Michele Bianchi, Stefania Fattori, Annalisa Modenese, Cristina Galli, Ivano Eberini, Pasquale Ferrante

**Affiliations:** a Laboratory of Molecular Virology, Department of Biomedical, Surgical and Dental Sciences, University of Milano, Milano, Italy; b Department of Biomedical Science for Health, University of Milano, Milano, Italy; c Department of Cardiology, Istituto Clinico Città Studi, Milano, Italy; d Interdepartmental Center of Diabetic Foot, Istituto Clinico Città Studi, Milano, Italy; e Laboratory of clinical analysis, Istituto Clinico Città Studi, Milano, Italy; f Department of Pharmacological and Biomolecular Sciences & DSRC, University of Milano, Milano, Italy; g Health Direction, Istituto Clinico Città Studi, Milano, Italy

**Keywords:** SARS-CoV-2, ORF6, stop codon, interferon, COVID-19 pathogenesis

## Abstract

Severe acute respiratory syndrome coronavirus 2 (SARS-CoV-2) was isolated from the oro/pharyngeal swabs of two Italian COVID-19 patients, physicians in a COVID-19 division hospital, with different courses of the disease. The complete genome sequences show that the two isolates belong to the B1.1 lineage, but contain a nucleotide mutation in the ORF6, leading to a stop codon and to the deletion of 6 amino acids in the C terminus. This deletion was unique, compared to the currently available sequences deposited in the GISAID and GenBank database. It did not affect the in vitro viral replication, neither the neutralizing activities of the patients' antibodies. Based on homology analysis with other Coronaviruses, the two isolated lacked the ORF6 aminoacidic portion responsible for the inhibition of the antiviral Interferon (IFN)-based host response. IFN seems to have a dual role of in SARS-CoV-2 infected patients: not only antiviral activity, but also a detrimental role in case of excessive production. A deletion in the SARS-CoV-2 ORF6 protein might have a specific, still unknown role in the viral pathogenesis.

## Letter

We report the complete sequence of SARS-CoV-2 strains originally isolated from nasopharyngeal swabs from two Italian physicians, affected with COVID-19, and working in a COVID-19 division of a general hospital of Milano, Italy.

Among the SARS-CoV-2 accessory proteins, ORF6, a small peptide of 61 aa, has been previously identified as possible type I interferon (IFN) antagonist [1,2], based also upon its similarity to the p6 encoded by SARS-CoV, which is able to prevent the host antiviral response, binding directly to the NPIPB3 protein, and preventing the activation of the STAT-1 pathway [3]. This is the first report of a SARS-CoV-2 sequence carrying a nucleotide mutation leading to a stop codon in the ORF6 coding region.

Nasopharyngeal swabs were obtained from two COVID-19 patients (#1 and #2) (Fondazione Ca’ Granda, Ospedale Maggiore, Milano, Italy protocol 456_2020, on May 2020). Protocols employed for the SARS-CoV-2 isolation, virus titration, RNA isolation from the cells surnatants and from the swabs, next generation sequencing (NGS), ORF 6 confirmatory PCR, SARS-CoV-2 antibodies neutralizing detection, viral growth curves, and database and homology searches are reported in the supplementary materials.

### Case reports

Patient #1: male, 51 years old, physician at the COVID-19 division of an Italian hospital, located in Milano, Lombardy, Italy. Starting on March, 4th, 2020, he had a daily assessment of 20 COVID-19 patients in the hospital. From March, 27th 2020, he experienced persistent pyrexia, and three days later the nasopharyngeal swab resulted positive for SARS-CoV-2. Hospitalization occurred on April, 2nd 2020: desaturation, thrombocytopenia, leukopenia, frank elevation of C reactive protein (21 mg/dl), elevation of interleukin 6 (64), and worsening of the chest x ray were observed. Treatment comprised continuous positive airway pressure, administration of hydroxychloroquine, azithromycin, Vitamin D, oral quercetin, subcutaneous heparin, anakinra. Discharge occurred on April, 24th 2020.

Patient #2: female, 48 years old, physician in the same hospital division of the patient#1. COVID-19 symptoms appeared on March, 24th 2020, with asthenia, cough and low-grade fever, with negative chest x-ray. A SARS-CoV-2 positive nasopharyngeal swab confirmed the diagnosis. She was subjected to home isolation for three weeks, with persistence of cough, altered taste, anosmia, no pyrexia. Treatment comprised paracetamol and paracodeine. SARS-CoV-2 negative swabs defined the healing on April, 23rd 2020. Risk factors of the patients were: cohabitation with COVID-19 husband and son, since March 11th 2020.

### Isolation and titration of SARS-CoV-2 from nasopharyngeal swabs

The first nasopharyngeal swabs were collected on March 30th (#1) and 24th (#2) and the Ct were 18 and 15, respectively. After inoculation, CPE were visible 72 h post infection, and isolation of SARS-CoV-2 was confirmed by RT-qPCR on the cell surnatants. RT-qPCR results, and viral titres were as follows: patient #1, Ct 15, (3.1 × 10^8^ copies/mL), 5.4 × 10^5^ PFU/ml; patient #2, Ct 12, (8.9 × 10^9^ copies/mL), 5.4 × 10^5^ PFU/ml. The sizes of the plaques were not different among the patients’ isolates.

### Next generation sequencing results

The nucleotide sequencing of the SARS-CoV-2 isolated strains was performed by an external facility (Eurofins Genomics, Konstanz, Germany), and results were deposited at Gen Bank, (SARS-CoV-2/human/ITA/Milan-ICCS-1-2/2020, MT956642.1), and at GISAID (EPI_ISL 584048, EPI_ISL 584049).

**Figure 1.**
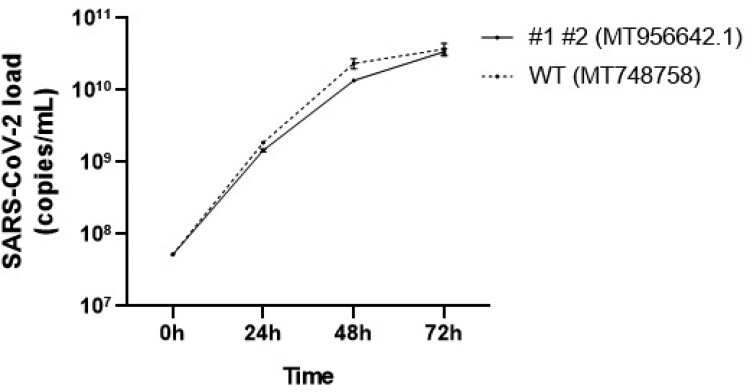
Growth cycle of ORF6 mutated strain (MT956642.1) and ORF6 wild type strain (MT748758). The viral load was quantified by RT-PCR in the surnatants of VERO-E6 infected cells, after 24, 48 and 72 h.

High quality sequencing reads were mapped to the reference SARS-CoV-2 genome isolate (Wuhan-Hu-1 (NC_045512.2)) (Table and Figure 1, Appendix).

Phylogenetic analysis performed with Pangolin v.1.14 assigned the strains to the lineage B1.1, based on the presence of the mutations at the positions 2881, 2882, and 2883, in the N gene. In the leader sequence, a high frequent mutation was present at the nucleotide 3037, while in the S gene, the point mutation 23403A > G, causing the missense amino acid change Asp614Gly in the Spike protein, was detected. A point mutation at the nucleotide 27367, in the ORF6 coding region was detected, leading to a stop codon in the protein, at the amino acid 55. (Table 2, Appendix).

### Confirmatory PCR and sequencing

Amplification and sequencing of the ORF6 gene was performed on RNA isolated from surnatants, and from nasopharyngeal swabs, collected for the patient #1 on March 30th, April 20th, 24th, 30th, May 05th, 08th, and 13th, and for the patient #2 on March 24th and April 14th. All the amplicons showed the mutation C > T at the nt. 27367 in the ORF6 (Figure 2, Appendix).

On the contrary, the mutation was not found in the RNA from 21 patients hospitalized from March 15th to 30th, in the COVID-19 unit, where the two physicians were working, and from one of patient #2’s relative.

### Viral growth curve

Growth curves of the ICCS 1–2 and UNIMI-1 (MT748758, EPI_ISL 584051) isolates were performed in Vero E6 cells, quantifying the viral load by RT-qPCR, in the surnatants after 24, 48 and 72 h. The two obtained curves were almost completely superimposable (Figure 1).

### Neutralizing antibody detection

Serum from patient #1 and #2 showed neutralization at dilution 1:4 and 1:32, respectively after 3 months, and 1:8 and 1: 64 after 6 months. No differences were observed among the results obtained using the two strains.

## Discussion

Based on the obtained results, we could observe that: (a) the point mutation was not an artefact due to the NGS assay, or to the passage of the SARS-CoV-2 in cells from non-human primates [4]; (b) the mutation found in ORF6 did not affect the immune protection of the patients, since both mutated and wild type ORF6 viral strains were neutralized by the patients’ sera or (c) the *in vitro* growth curves; (d) the mutation did not derive from a virus infecting other hospitalized patients or one of the patient #2’s relative. The mutation might be derived from an intrahost process of variation, that is a common phenomenon in Coronaviruses, due to the error- prone replication, and to the discontinuous RNA synthesis, resulting in genomic rearrangement and/or recombination [5]. The generation of quasispecies diversity upon intrahost variations might be the cause of persistent infection in the host, since it provides the virus a chance to evolve [6].

The phylogenetic relationship between SARS-CoV-2 ORF6 protein and the identified ortholog ones (D2DJW9, P59634, B8Q8U2, Q3I5J1, Q0Q471, Q3LZX8, A0A0K1Z0N6, Q692D9, Q5DIC0, A0A0U1WHG3, E0XIZ7), SARS coronavirus BJ182-4 Accessory protein 6 and Bat coronaviruses Non-structural or Accessory protein 6, allowed us to advance some hypotheses.

In SARS-CoV infected cells, ORF6 protein localized on the rough endoplasmic reticulum/Golgi membrane [3]. It was shown that SARS-CoV ORF6 inhibited IFN-γ production, and the IFN- γ signalling, by inhibiting the translocation of STAT1 in the nucleus [7]. SARS-CoV-2 ORF6 was identified as type I IFN antagonist *in vitro*, together with ORF 8, N [1], nsp13, 14, 15 [2]. Deletion of the SARS-CoV-2 ORF6 gene, on the contrary, resulted in induction of IFN production [3].

The biochemical role of SARS-CoV-2 ORF6 protein, connected with the block of STAT1 nuclear translocation in response to IFN signalling, can also be hypothesized by homology with a statistically significant level of confidence (E-value 2.5e-28, BLAST search) from the experimental evidence on SARS-CoV ORF6 protein. The same inference-based procedure suggested that this mechanism could be mediated by the molecular recognition of KPNA2 [3]. It was reported that a recombinant SARS-CoV, in which the ORF 6 was removed, cannot control the localization of KPNA2 in the ER/Golgi membrane, favouring STAT1 import at nuclear level [3]. Additionally, the residues 54–63 of SARS-CoV ORF6 protein were critical for disrupting nuclear import processes, suggesting the molecular basis for the impact of the identified mutation in our patients, and this is exactly the lacking missing C-terminal part of the ORF6 protein in our isolates. Indeed, IFN has been recommended as potential therapeutic drugs to prevent and treat SARS-CoV-2 infection [2].

However, in COVID-19 disease, increased levels of IFN-γ were associated with pulmonary inflammation and extensive lung damage, both hallmarks of deterioration [8,9]. Along with IL-6, IFN-γ has been a reliable indicator of COVID-19 patient deterioration and intensive care unit admission [10]. Excessive and prolonged IFN expression might lead to proinflammatory responses and may aggravate SARS-CoV-2 infection by disrupting the lung epithelial barrier [11]. Thus, IFN role in the host response of COVID-19 patients shows a still unknown, dual significance.

As secondary conclusion, we could also observe that in our patients, a more severe disease leads to higher antibodies titre, and also that in both patients the immune response against the virus increased over the time. Both the observations might be important in the light of the strong utility of the SARS-CoV-2 vaccination [12].

In this still uncertain scenario of the COVID-19 pathogenesis, the isolation of new virus variants, employing possible, even if hypothetical, different mechanism of pathogenesis could be helpful in the study of innovative therapeutic strategies.

## Supplementary Material

appendices_orf6rev2_cleancopy.docx

supplementary_data_orf6rev2_cleancopy.docx
