# Supplementary material for: Isolation of SARS-CoV-2 strains carrying a nucleotide mutation, leading to a stop codon in the ORF 6 protein
Source: Emerg Microbes Infect. 2021 Feb 18;10(1):252–5. doi: 10.1080/22221751.2021.1884003 (PMC7894437; doi:10.1080/22221751.2021.1884003)
Supplement: appendices_orf6rev2_cleancopy.docx [file TEMI_A_1884003_SM2153.docx]

**Appendices**

Table 1: SARS-CoV-2 screening by NGS

| **Sample Name** | **Total Read (million)** | **Mapped reads** | **Genome coverage** | **Average base coverage** | **Number of variants detected** |
| --- | --- | --- | --- | --- | --- |
| # 1 | 16.3 M | 98.30% | 99.80% | 68733 X | 7 |
| # 2 | 19.1 M | 98.60% | 99.80% | 81492 X | 7 |

Table 2: Variants found in the SARS-CoV-2 isolates, compared to the Wuhan-Hu-1 isolate (NC_045512.2)

| **Nt position** | **Mutation** | **Gene** | **Variant Type** | **Amino acid change** | **#1 coverage** | **#1 frequency** | **#2 coverage** | **#2 frequency** |
| --- | --- | --- | --- | --- | --- | --- | --- | --- |
| 3037 | C>T | orf1ab | synonim | Phe924Phe | 4653 | 99.94% | 5282 | 99.92% |
| 14408 | C>T | orf1ab | missense | Pro4715Leu | 6404 | 99.91% | 6476 | 99.85% |
| 23403 | A>G | S | missense | Asp614Gly | 6858 | 99.97% | 6909 | 99.96% |
| 27367 | C>T | ORF6 | Stop | Gln56* | 7144 | 99.87% | 7270 | 99.81% |
| 28881 | G>A | N | missense | Arg203Lys | 6469 | 99.92% | 6806 | 99.99% |
| 28882 | G>A | N | synonim | Arg203Arg | 6432 | 99.95% | 6814 | 100% |
| 28883 | G>C | N | missense | Gly204Arg | 6474 | 99.95% | 6861 | 99.94% |

**Figure 1
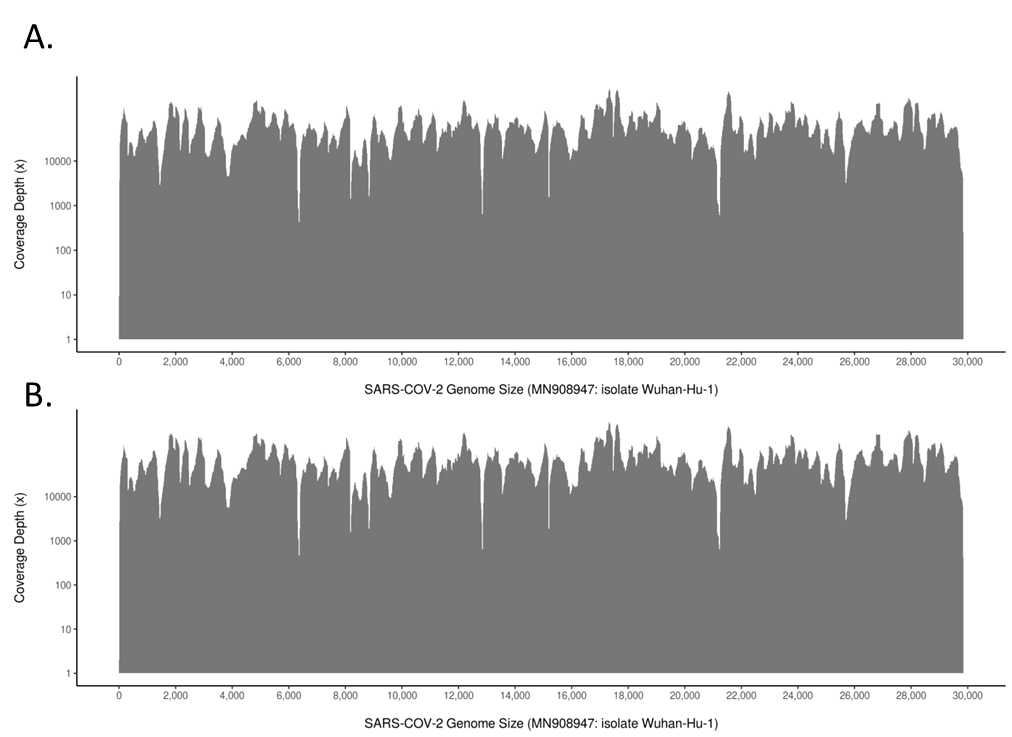
**

**Figure 2**

**
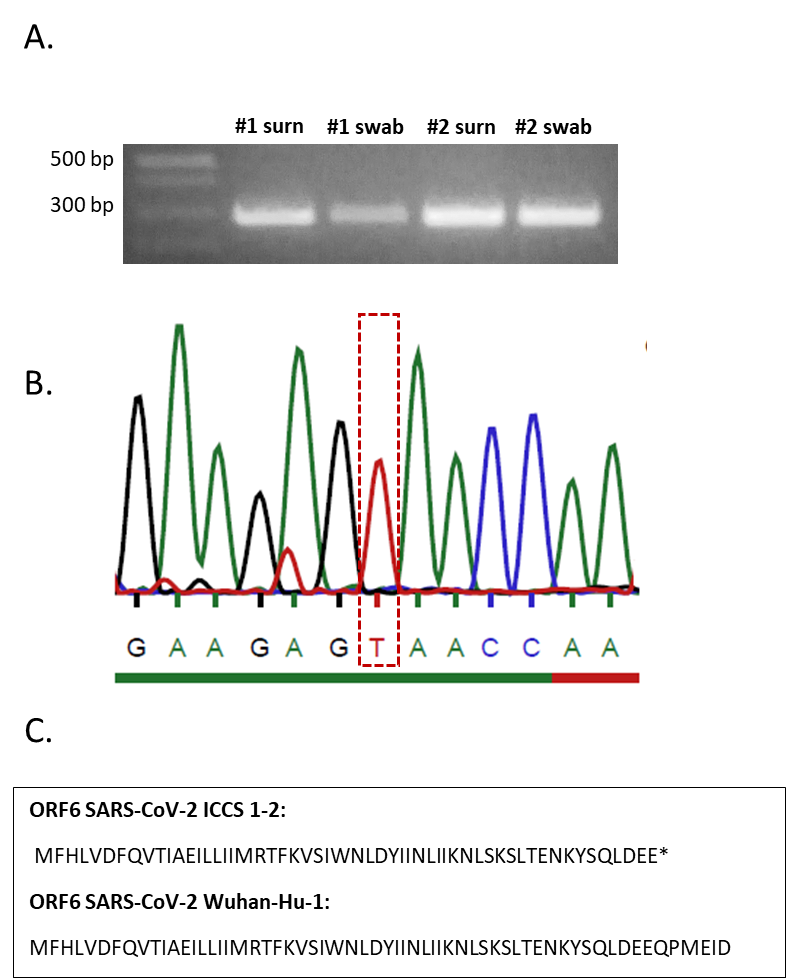
**

**Figure captions**

**Figure 1:** SARS-CoV-2 genome coverage of RNA sequencing of isolates from patient #1 (panel A), and patient #2 (panel B). X-axis shows the size of SARS-CoV-2 genome in base pair and y-axis shows the coverage depth (number of sequencing reads covering at each position of the genome)

**Figure 2:** SARS-CoV-2 ORF6 point nucleotide mutation was further verified by RT-PCR showing the right size amplicon size, (A) and by Sanger sequence (B). Panel (C) shows the mutated protein sequence (MT956642.1), aligned with the Wuhan-Hu-1 reference sequence. (NC_045512.2).
