# Supplementary material for: Isolation of SARS-CoV-2 strains carrying a nucleotide mutation, leading to a stop codon in the ORF 6 protein
Source: Emerg Microbes Infect. 2021 Feb 18;10(1):252–5. doi: 10.1080/22221751.2021.1884003 (PMC7894437; doi:10.1080/22221751.2021.1884003)
Supplement: supplementary_data_orf6rev2_cleancopy.docx [file TEMI_A_1884003_SM2152.docx]

**Supplementary materials:**

**Material and Methods**

***Virus isolation and growth***

Nasopharyngeal swabs were obtained from two COVID-19 patients (patient #1 and #2), upon approval of the Local Ethical Committee and signature of the informed consent (Fondazione Ca’ Granda, Ospedale Maggiore, Milano, Italy approved the protocol 456_2020, on May 2020).

SARS-CoV-2 isolation was conducted by inoculating 500 µl of the viral transport medium on Vero cells (Monkey Kidney Epithelial Cells, ATCC CCL81), maintained in DMEM medium, supplemented with 10% heat-inactivated fetal bovine serum (FBS), 2 mM glutamine, 100 units/ml penicillin, 100 µg/ml streptomycin and 2.5 µg/ml Amphotericin B, at 80%-90% confluence, in a T25 flask. Virus absorption process was allowed for 2 hours, at 37°C, after which the inoculum was completely removed, and DMEM medium with 10% FBS was added. The infection was maintained at 37°C, 5% CO_2_, until the appearance of cytopathic effects (CPE) was evident.

***Virus titration***

Viral titer was determined by plaque assay. Vero cells were seeded into 6-well plates at a density of 5×10^5^ cells/well and incubated for 24 hours at 37 °C, 5% CO2. Cells infected surnatants (500 µl), serially diluted from 10^1^ to 10^9^, were added to six plate wells for 2 hours at 37°C, 5% CO_2_. Virus inoculum was removed, and the cells were covered with 0.3% agarose dissolved in DMEM, and incubated for 72 hours at 37 °C, 5% CO_2_. Cells were then fixed with 4% formaldehyde solution and, after agarose removal, stained with methylene blue. Plaques were counted and results were expressed as Plaque Forming Unit (PFU)/mL.

***RNA Isolation***

RNA was isolated from 150 µl of infected cells medium or from the viral transport medium of the nasopharyngeal swabs, using the Nucleospin RNA virus kit (Macherey-Nagel, Germany), following the manufacturer’s protocol. Viral copy numbers in the samples were quantified via specific qRT-PCR, targeting the N1 gene, following the CDC protocol, using the 7500 Gene Systems (Applied Biosystems, USA).

***Next Generation Sequencing (NGS)***

RNA was extracted from the cell supernatants, as previously described, and cleaned up, using the RNeasy MinElute Cleanup Kit (Qiagen), following the manufacturer’s instructions. One-hundred nanograms of RNA were maintained in 1/10 volume 3 M sodium acetate (pH 5.2), 1/50 volume glycogen (5mg/ml), and 3 volumes of ice cold 100% ethanol, and sent to an external facility, that uses the Illumina platform for NGS (Eurofins Genomics, Konstanz, Germany), and fills in a final screening report.

***Viral Growth Curve***

Vero E6 [C1008] cells were seeded into 6-well plates at a density of 5×10^5^ cells/well and incubated for 24 hours at 37 °C, 5% CO_2_. The mutated and the wild type ORF6 SARS-CoV-2 were inoculated on the cells, at MOI 0.01, for 2 hours, at 37 °C, 5% CO_2_. After the virus absorption process, the inoculum was completely removed, the cells were washed with PBS, and DMEM medium with 10% FBS was added. The infection was maintained for 24, 48 or 72 hours at 37 °C, 5% CO_2_. After the infection time, RNA was isolated from surnatant as previously described and RT-qPCR for N1 gene was performed.

***Confirmatory PCR***

The presence of the ORF6 nucleotide point mutation found in the two isolates was verified by amplification of the complete ORF6 genomic region, by means of one-Step RT-PCR, on the RNA isolated from the cell surnatants and from nasopharyngeal swabs. The primer sequences were as follows: ORF-6 Fw, 27149-CCATTCCAGTAGCAGTGACAATA -27172, and ORF-6 Rev, 27420-GCTCACAAGTAGCGAGTGTTAT-27442.

The reaction mix was prepared in a final volume of 50 µl: 25 µl of 2x Reaction mix, 2 µl of SuperScript III RT/Platinum Taq Mix (ThermoFisher Scientific, USA), 10 µM of each primer, and 5 µl of RNA samples. The reaction steps were: 1 cycle of 50°C for 15 min, and 95°C for 5 min. Then, 40 cycles of 95°C for 15 sec, 60°C for 30 sec, and 68°C for 40 sec. Finally, the last step was performed at 68°C for 5 min. Amplification products (length 292 bp) were analyzed on a 2% agarose gel in a 0.5× TBE buffer. The PCR products were purified using the QIAquick Gel Extraction Kit (Qiagen, Germany). The automatic sequencing was performed by the Eurofins company (Milan, Italy), according to the Sanger method.

***SARS-CoV-2 neutralizing antibody detection***

Serum samples were collected after 3 and 6 months, and maintained at -80°C until the use. The sera were heated at 56°C for 45 minutes to inactivate the complement proteins, 2-fold serially diluted (from 1:2 to 1:1024), and incubated with 100 Tissue Culture Infectivity Dose (TCID) 50 of the viruses isolated from the patients, or with the strain SARS-CoV-2 UNIMI-1 (Accession number: MT748758, EPI_ISL 584051), previously isolated in the laboratory, for 1 hour, at 37°C. Then 30 µl of virus-serum mix was inoculated into Vero cells previously plated in 96 wells, for 2 hours at 37°C, after which 170 µl of complete medium was added. Each dilution was repeated in 5 wells, in two independent experiments. After 3 days, the plates were inspected by an inverted optical microscope. The highest serum dilution that protected 100% of the cells from CPE was taken as the neutralization titer.

***Database and homology search***

Information about SARS-CoV-2 ORF6 protein (P0DTC6) was collected from the specific section of the UniProt database (https://covid-19.uniprot.org/uniprotkb/P0DTC6), which was also used for an orthology search with BLAST in the UNIPROTKB.
